# Supplementary material for: Betacoronaviruses Differentially Activate the Integrated Stress Response to Optimize Viral Replication in Lung-Derived Cell Lines
Source: Viruses. 2025 Jan 16;17(1):120. doi: 10.3390/v17010120 (PMC11769277; doi:10.3390/v17010120)
Supplement: Supplementary file 1 [file viruses-17-00120-s001.zip › viruses-3416691-supplementary.pdf]

Table S1. Antibodies				
**Primary Antibody**	**Antibody Species**	**Blocking Buffer**	**Dilution**	**Catalog Number**
PERK	rabbit	5% milk	1:1000	Cell Signaling Technology 3192S
pPKR (phospho-T446) [E120]	rabbit	5% milk	1:1000	Abcam 32036
PKR (D7F7)	rabbit	5% milk	1:1000	Cell Signaling Technology 12297S
p-eif2α (S51)	rabbit	5% milk	1:1000	Cell Signaling Technology 9721S
eif2α	rabbit	5% milk	1:1000	Cell Signaling Technology 9722S
GADD34	rabbit	5% milk	1:600	10449-1-AP (Protein Tech)
CReP	rabbit	5% milk	1:1000	14634-1-AP (Protein Tech)
GAPDH (14C10)	rabbit	5% milk	1:2000	Cell Signaling Technology 2118S
SARS-CoV-2 N	rabbit	5% milk	1:2000	GTX135357 (Gentex)
MERS-CoV N	mouse	5% milk	1:2000	40068-MM10 (Sino Biological)
HCoV-OC43 N	rabbit	5% milk	1:2000	40643-T62 (Sino Biological)
**Secondary Antibody**				
goat anti-rabbit IgG	HRP linked	same as primary	1:3000	Cell Signaling Technology 7074S
goat anti-mouse IgG	HRP linked	same as primary	1:3000	Cell Signaling Technology 7076S

**Table S2. Oligonucleotide primers**		
**Target**	**Forward Primer (5' to 3')**	**Reverse Primer (5' to 3')**
ATF3	CGCTGGAATCAGTCACTGTCAG	CTTGTTTCGGCACTTTGCAGCTG
DDIT3 (CHOP)	GGTATGAGGACCTGCAAGAGGT	CTTGTGACCTCTGCTGGTTCTG
GADD34	AGCCACGGAGGATAAAAGAACA	CTGAACGATACTCCCAGGACC
CReP	TGAGGATTGGGATGAGGAAG	TCTGGCAGCAGTCTGAATTG
18S rRNA	TTCGATGGTAGTCGCTGTGC	CTGCTGCCTTCCTTGAATGTGGTA

Fig. S1. CC_{50} and EC_{50} of salubrinal in A549 cells. A) $A549^{ACE2}$ cells were treated with the indicated concentration of salubrinal for 48 hours. After incubation, cellular viability was measured using Cell Titer-Glo 2.0. Percent viability was determined after subtracting the background and normalizing to the untreated control. B) $A549^{ACE2}$ cells were infected with HCoV-OC43 at an MOI of 0.1 PFU/cell and subsequently treated with the indicated concentration of salubrinal. At 48hpi, supernatants were collected and titered by plaque assay. The EC_{50} was determined by normalizing each condition to the untreated control. Statistics were calculated using one-way ANOVA. ** = $p < 0.01$; **** = $p < 0.0001$.

Figure S2: Salubrinal treatment reduces WT MERS-CoV and a MERS-CoV mutant virus replication. $A549^{DPP4}$ cells were infected with MERS-CoV WT or MERS-CoV $nsp15^{mut}/\Delta NS4a$ at MOI = 0.1 PFU/cell. Immediately following infection, cells were left untreated or treated with 20μM salubrinal for the course of the infection. Infectious virus was quantified by plaque assay of supernatants collected from infected cells. Statistics by 2-way ANOVA. * = $p < 0.05$; ** = $p < 0.01$; *** $p < 0.001$; **** = $p < 0.0001$.

Figure S3: GADD34 knockout reduces translational recovery. $A549^{ACE2}$ single-cell clones with GADD34 knocked out (B and C) or a scramble guide RNA (sgCtrl, A) were treated with 1μM thapsigargin (Tg) or mock treated. At the indicated times, 10μg/mL puromycin was added to the media for 10 minutes before cells were lysed and whole-cell lysates collected. Western immunoblots were performed for the indicated proteins or for puromycin. Cells transduced with scrambled sgRNA (sgCtrl) show rapid GADD34 accumulation and a resumption of translation after 2 hours of Tg treatment. ΔGADD34 cells fail to produce GADD34 protein or restart translation.

Figure S1

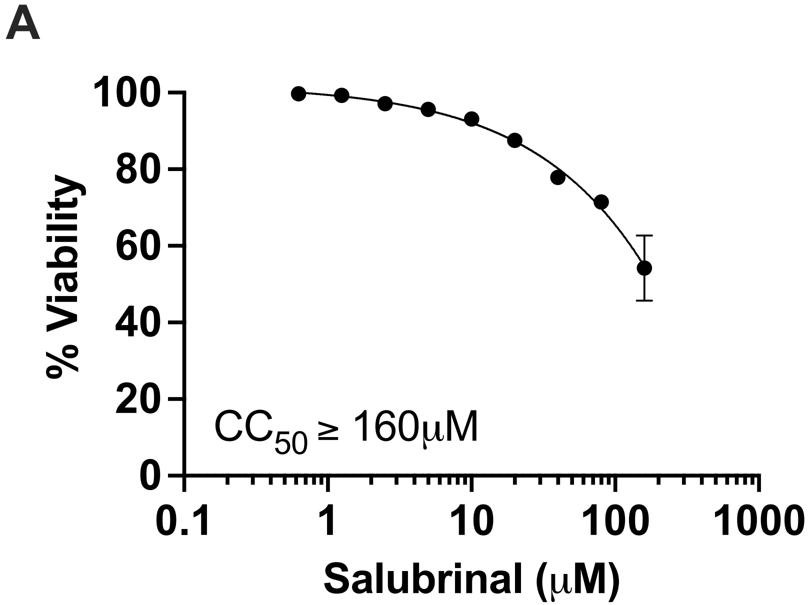
A
100
80
60
40
20
0
% Viability
CC50 ≥ 160μM
0.1
1
10
100
1000
Salubrinal (μM)

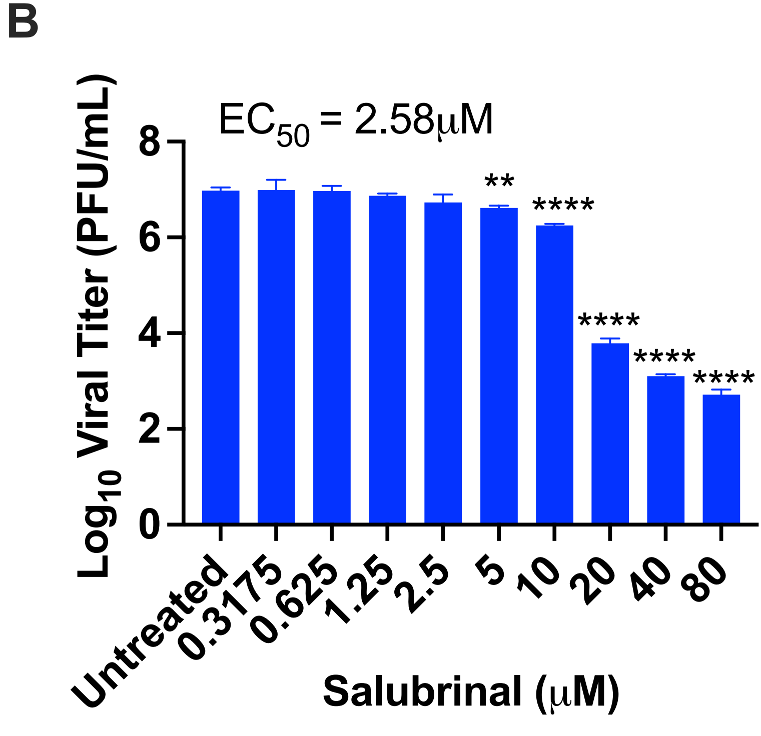
B
EC50 = 2.58μM
8
6
4
2
0
Log10 Viral Titer (PFU/mL)
**
****
****
****
****
Untreated
0.3175
0.625
1.25
2.5
5
10
20
40
80
Salubrinal (μM)

Figure S2

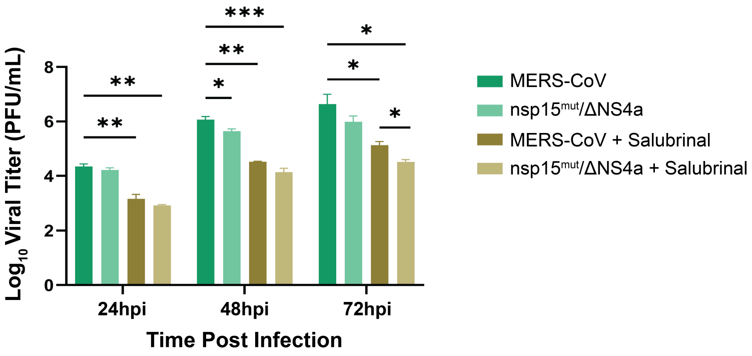

***
**
**
*
*
**
*
*
8
6
4
2
0
Log10 Viral Titer (PFU/mL)
24hpi
48hpi
72hpi
Time Post Infection
MERS-CoV
nsp15mut/ΔNS4a
MERS-CoV + Salubrinal
nsp15mut/ΔNS4a + Salubrinal

Figure S3

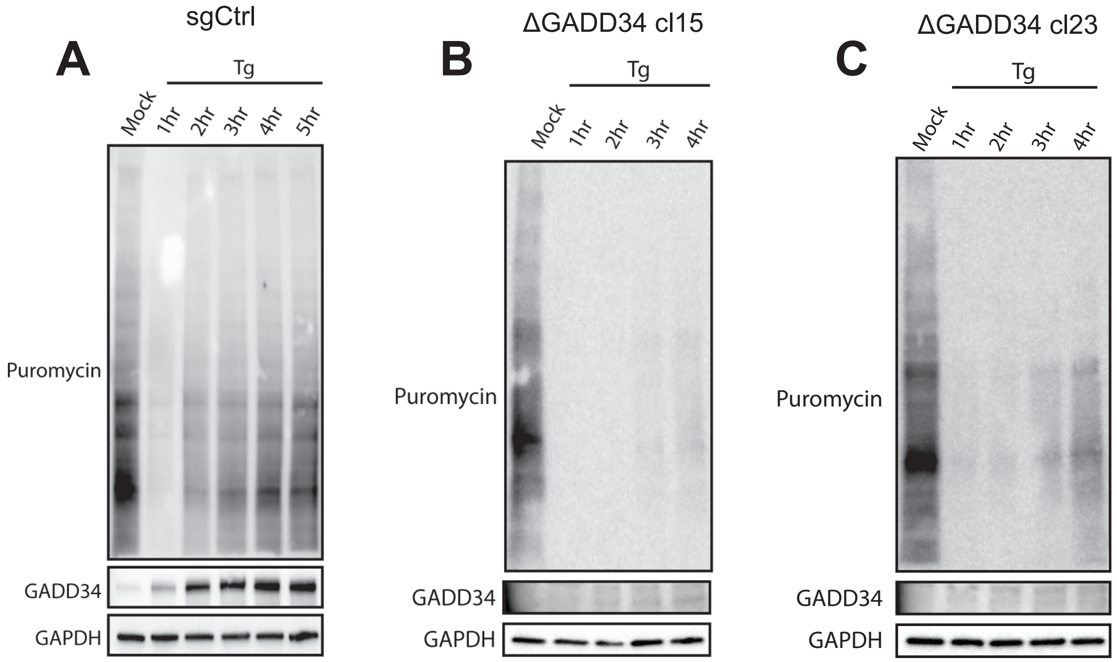

A
sgCtrl
Tg
Mock
1hr
2hr
3hr
4hr
5hr
Puromycin
GADD34
GAPDH
B
ΔGADD34 cl15
Tg
Mock
1hr
2hr
3hr
4hr
Puromycin
GADD34
GAPDH
C
ΔGADD34 cl23
Tg
Mock
1hr
2hr
3hr
4hr
Puromycin
GADD34
GAPDH
